# Mild Ventriculomegaly, Ventricular Asymmetry, and Posterior Fossa Variants: A Narrative Review of Borderline Fetal Brain Findings

**DOI:** 10.3390/diagnostics16193144

**Published:** 2026-09-27

**Authors:** Iulian Gabriel Goidescu, Georgiana Nemeti, Adelina Staicu, Mihai Surcel, Dan Boitor, Cerasela Mihaela Goidescu, Ioana Cristina Rotar, Gheorghe Cruciat, Daniel Muresan

**Affiliations:** 1 Obstetrics and Gynecology I, Mother and Child Department, University of Medicine and Pharmacy “Iuliu Hațieganu”, 400006 Cluj-Napoca, Romania; 2 Obstetrics Gynecology I, Emergency County Hospital, 400006 Cluj-Napoca, Romania; 3 Department of Internal Medicine, Medical Clinic I—Internal Medicine, Cardiology and Gastroenterology, University of Medicine and Pharmacy “Iuliu Hațieganu”, 400006 Cluj-Napoca, Romania

**Keywords:** fetal neurosonography, mild ventriculomegaly, non-dilated ventricular asymmetry, mega cisterna magna, Blake’s pouch cyst, prenatal ultrasound, fetal MRI, neurodevelopmental outcome, parental counseling

## Abstract

Recent advances in fetal neurosonography have markedly improved the detection of subtle central nervous system findings that were previously identified less frequently. Mild ventriculomegaly (MV), non-dilated ventricular asymmetry (NDVA), mega cisterna magna (MCM), and Blake’s pouch cyst (BPC) are increasingly encountered during routine prenatal ultrasound and, in the absence of associated genetic or infectious abnormalities, are commonly regarded as borderline conditions or anatomical variants. These findings frequently pose challenges in prognostic evaluation and in the counseling of expectant parents. The objective of this review is to summarize current evidence on the detection, characterization, and clinical management of these four entities and to provide a practical framework for evidence-based parental counseling. This narrative review examines the diagnostic approach to MV, NDVA, MCM, and BPC using both ultrasound and fetal magnetic resonance imaging (MRI). A uniform analytic framework is applied to each entity, addressing in turn etiology, sonographic assessment, the complementary role of MRI, genetic evaluation, and prognosis with counseling considerations. Representative ultrasound and fetal MRI images from a tertiary referral center are used for illustrative purposes. Across all four entities, fetal MRI provides complementary information in selected cases, while genetic testing and serological screening may help exclude chromosomal or infectious etiologies. Neurodevelopmental outcomes are broadly favorable when the finding is truly isolated, but the prognostic literature is limited by small samples, heterogeneous definitions of “isolated,” short follow-up, and unstandardized neurodevelopmental assessments. Common themes across these entities are synthesized into a staged framework intended to support evidence-based parental counseling regarding the expected neurodevelopmental outcomes of these fetuses.

## 1. Introduction

Antenatal ultrasound assessment of the fetal brain is a cornerstone of prenatal evaluation, frequently identifying a spectrum of findings that may be considered normal variants or borderline. Among the most commonly encountered are mild ventriculomegaly (MV), non-dilated ventricular asymmetry (NDVA) and posterior fossa abnormalities such as mega cisterna magna (MCM) and Blake’s pouch cyst (BPC) [1].

In contrast to major fetal brain malformations, these subtle or borderline findings pose significant challenges in determining the appropriate extent of prenatal investigations. When such anomalies are detected, counseling typically includes the recommendation to complement ultrasonography with fetal magnetic resonance imaging and to consider invasive genetic testing. Nevertheless, accurately predicting postnatal outcomes remains difficult, and the optimal strategy for postnatal follow-up, particularly regarding long-term neurocognitive development, has yet to be clearly defined [2,3].

Physiological human brain development is characterized by inherent anatomical and volumetric asymmetries between cerebral hemispheres and lateral ventricles, which contribute to functional specialization and sex-related behavioral differences. The cerebellum serves as the primary coordinating center for motor activity, playing a central role in the execution and coordination of movements [4,5,6]. In addition, it contributes to spatial navigation and musical learning and functions as a modulator of cognitive, emotional, and autonomic areas [6].

Regarding posterior fossa anomalies such as MCM and BPC, they can be readily identified on ultrasound but are sometimes difficult to differentiate from each other and from more serious pathologies such as the Dandy–Walker continuum (DWC) [7].

The identification of such findings often triggers extensive prenatal evaluation, including repeated targeted neurosonographic examinations, maternal serological testing, fetal echocardiography, and genetic investigations. These may involve formal genetic counseling, invasive diagnostic procedures such as amniocentesis, and chromosomal microarray analysis (CMA) [1].

Among antenatally detected cerebral findings, MV, NDVA and posterior fossa anomalies such as MCM and BPC—particularly in the absence of associated genetic abnormalities—are generally associated with a favorable neonatal neurological outcome. However, the reassuring message conveyed by much of the published literature must be interpreted with caution: many studies are limited by small sample sizes, short follow-up periods, absence of standardized neurodevelopmental assessment tools, and referral bias. These methodological shortcomings warrant careful consideration when counseling families.

The aim of this narrative review is to summarize current evidence on the detection, evaluation, and management of these minor antenatally identified cerebral anomalies, illustrated by representative ultrasound and fetal MRI images from a tertiary referral center. This review is structured around a uniform analytic framework: each of the four entities—mild ventriculomegaly, non-dilated ventricular asymmetry, mega cisterna magna, and Blake’s pouch cyst—is examined in turn with respect to etiology, sonographic assessment, the complementary role of magnetic resonance imaging, genetic evaluation, and prognosis with counseling considerations. The four entity-specific sections are followed by an integrated discussion that draws out the themes common to all four and proposes a practical framework for clinical practice. Within this structure, we discuss the comparative value of ultrasound and MRI for each entity; critically evaluate the role and diagnostic yield of genetic testing across modalities (karyotype, chromosomal microarray analysis, and whole-exome sequencing); provide a balanced appraisal of the prognostic literature, explicitly acknowledging its limitations; and offer practical guidance for parental counseling. These four entities are grouped here on clinical rather than nosological grounds, but the grouping is not arbitrary: all four are borderline findings of the fetal cerebrospinal fluid spaces—the lateral ventricles in the case of MV and NDVA, and the posterior fossa compartments derived from the fourth ventricle and Blake’s pouch in the case of MCM and BPC. In each, the diagnostic question is the same: whether a borderline enlargement or asymmetry of a fluid space represents a normal variant or the earliest expression of an obstructive, destructive or maldevelopmental process. In each, the same evaluation cascade follows—serial biometry of the fluid space, targeted MRI for associated parenchymal anomaly, genetic testing, and screening for congenital infection. Borderline findings whose clinical logic differs are therefore not included: choroid plexus cysts, which function principally as a soft marker for trisomy 18, and persistent midline cavities such as the cavum vergae and the cavum veli interpositi. The synthesis that follows is narrative and is intended to summarize and interpret the available evidence; it does not constitute a clinical practice guideline, and the practical suggestions offered should be read as expert opinion calibrated to the strength of the underlying data.

## 2. Materials and Methods

This narrative review was based on a structured search of PubMed/MEDLINE, Scopus and Web of Science, last updated on 25 August 2026. Because the four entities are individually uncommon and much of the foundational prognostic evidence predates the last decade, no date restriction was applied, so that seminal cohorts and series would not be excluded. The complete search strings for each database are provided in Supplementary Table S1. In brief, population terms (“fetal”, “fetus”, “prenatal”, “antenatal”) were combined with entity terms (“ventriculomegaly”, “ventricular asymmetry”, “mega cisterna magna”, “Blake’s pouch cyst”, “posterior fossa”) and with terms for the domains of interest (“magnetic resonance imaging”, “chromosomal microarray”, “exome sequencing”, “genome sequencing”, “congenital infection”, “neurodevelopmental outcome”). The reference lists of key articles and of current society guidelines were hand-searched.

Records were restricted to publications in English. Studies were considered eligible if they reported original clinical data on one or more of the four entities in fetuses or children in whom the finding had been identified antenatally—including case reports, case series, retrospective and prospective cohorts, systematic reviews and meta-analyses—and if they addressed at least one of the following: diagnostic criteria or measurement technique, associated anomalies, genetic yield, congenital infection, natural history, or neurodevelopmental outcome. Studies were excluded if the finding was not identified antenatally, if the entity of interest could not be separated from other ventricular or posterior fossa pathology in the reported data, or if no extractable outcome was reported. Because the total published experience for these entities is small, no minimum sample size was applied, and case reports were retained. Where a case report is the sole source for a statement, this is indicated in the text and in Table 1, Table 2, Table 3 and Table 4.

Study selection was performed independently by 2 clinicians with disagreements resolved by discussion and, where necessary, by a third professional. Duplicates across databases were removed manually before screening.

The supporting literature is heterogeneous and predominantly observational. No formal risk-of-bias or quality-appraisal instrument was applied, and we state this as a deliberate limitation rather than an oversight: STROBE is a reporting guideline rather than a risk-of-bias tool, and the Newcastle–Ottawa Scale is validated for comparative observational studies, whereas a substantial part of the evidence base for these four entities consists of single-arm case series and case reports for which it yields no interpretable score. Instead, for each entity, the relevant studies were tabulated against a common set of dimensions—study design, sample size, the definition of “isolated” used, imaging confirmation, the neurodevelopmental assessment method and follow-up duration, and the main outcome—so that the evidence base could be compared directly and its limitations made explicit (Table 1, Table 2, Table 3 and Table 4). Priority in the narrative synthesis was given to the largest cohorts, to systematic reviews and meta-analyses, and to current society guidelines (ISUOG—International Society of Ultrasound in Obstetrics and Gynecology, SMFM—Society for Maternal-Fetal Medicine, WAPM—World Association of Perinatal Medicine), with case reports and small series retained where they illustrate a specific diagnostic or prognostic point.

Because the review was designed as a narrative rather than a systematic synthesis, the number of records identified and screened at each stage was not logged prospectively, and a PRISMA flow diagram is therefore not presented; this is acknowledged as a limitation in Section 8.

### Operational Definition of an Isolated Finding

Because the prognostic literature for all four entities is framed in terms of “isolated” findings, and because the term is used inconsistently across studies, we set out at the outset the definition used throughout this review. Isolation is not a single state but a graded one, and the level attained should be stated explicitly whenever a prognosis is quoted.

Level 1—isolated on ultrasound. No additional CNS or extra-CNS anomaly on dedicated multiplanar neurosonography and a detailed anatomical survey, including fetal echocardiography where indicated. This is the minimum requirement and the level at which most counseling in practice takes place.

Level 2—isolated after advanced imaging. Level 1, with no additional anomaly demonstrated on fetal MRI.

Level 3—isolated after complete etiologic evaluation. Level 2, with normal genetic evaluation (karyotype and/or chromosomal microarray, with NIPT as a screening alternative where invasive testing is declined), negative targeted screening for congenital infection, and persistence of isolation on serial examination.

This graded scheme is used deliberately in place of a single restrictive definition. Requiring genetic testing and MRI before a finding may be called isolated would exclude from that category the many pregnancies in which invasive testing is declined or fetal MRI is not available, even where no additional abnormality is demonstrable—and these are precisely the pregnancies in which counseling is nonetheless required. Throughout this review, and in Table 1, Table 2, Table 3 and Table 4, we indicate the level of isolation on which each reported outcome rests, since prognostic figures derived from Level 3 cohorts cannot be transferred without qualification to a fetus assessed only at Level 1.

Isolation also remains provisional. It depends on gestational age at assessment, on image quality and operator experience, and on the possibility that an associated finding declares itself later in gestation or only after birth.

## 3. Mild Ventriculomegaly (MV)

Fetal ventriculomegaly represents the prenatal ultrasound detection of cerebral ventricles with atrial diameters of 10 mm or more, with an estimated prevalence of approximately 1–2 per 1000 births. The mild form, on which this review focuses, is defined by an atrial diameter of 10–12 mm. It represents one of the most frequently identified fetal cerebral anomalies on ultrasound examination, most commonly detected between 18 and 24 weeks of gestation [8,9], and accounts for approximately 23% of central nervous system (CNS) malformations, with more than half of affected fetuses presenting associated anomalies outside the CNS [9].

### 3.1. Etiology

The pathogenesis of the various forms of ventriculomegaly is associated with an estimated 5% risk of aneuploidy (predominantly trisomy 21), a 10–15% risk of other genetic abnormalities, and approximately a 5% risk of congenital infections [9,10]. MV may be associated with a range of underlying cerebral abnormalities, most commonly including complete or partial agenesis of the corpus callosum, aqueductal stenosis, cerebellar malformations, abnormalities of cortical development, Chiari malformation, and Dandy–Walker syndrome [11,12,13,14].

The prevalence of intra-amniotic infection as an etiological factor has been hypothesized, with an approximately 1.4% incidence, with cytomegalovirus and toxoplasmosis most frequently implicated [15,16]. Other viral infections, including rubella, parvovirus B19, Zika virus, and herpes viruses, have also been associated with ventriculomegaly, triggering infectious screening [15,16,17]. In many healthcare systems, including our own, maternal TORCH serology is already performed as part of routine first-trimester antenatal surveillance, and these results should be retrieved and interpreted when MV is detected. At the time of diagnosis, we suggest maternal CMV and toxoplasma serology as first-line testing, with additional serology directed by exposure history, maternal symptoms, or associated sonographic findings such as intracranial calcifications, hepatosplenomegaly, growth restriction or effusions, since untargeted extension of serological testing carries a low diagnostic yield and a substantial rate of uninterpretable results [18]. Amniotic fluid PCR analysis is reserved for cases in which maternal serology is positive or equivocal, or in which the sonographic phenotype is suggestive despite negative serology [18].

### 3.2. Neurosonography Anatomic Landmarks and Assessment Pattern

Lateral ventricle assessment is a mandatory step during fetal neurosonography. The lateral ventricles are paired C-shaped cavities, each consisting of a body and an atrium, together with frontal, temporal, and occipital horns (Figure 1). Development commences after neural tube closure and proceeds rapidly; by 21 weeks the horns are clearly delineated, and by 31 weeks the ventricles assume an essentially adult configuration [19,20,21].

The ≥10 mm threshold, originally established by Cardoza and colleagues, reflects the relative stability of the atrial diameter across the second and third trimesters, with a normal mean of approximately 7.6 mm [22]. Measurement technique matters: Cardoza’s original outer-to-outer mean was 7.6 mm, whereas inner-to-inner techniques (now standard per ISUOG) yield a lower mean around 6.5–6.7 mm [22,23].

### 3.3. Ultrasound Assessment

The atrial diameter (AD) should be measured in the axial transventricular plane at the level of the frontal horns and the cavum septi pellucidi, with calipers on the inner margins of the atrium, perpendicular to the long axis of the lateral ventricle [24] (Figure 2a). VM is classified as mild (AD 10–12 mm), moderate (13–15 mm), and severe (>15 mm) [25].

The atrial diameter remains relatively stable between 15 and 40 weeks, and ventriculomegaly generally cannot be reliably diagnosed before 15 weeks [26]. Mild ventriculomegaly, with an atrial diameter below 12 mm and a head circumference above the 50th percentile, is increasingly regarded as a normal developmental variant [26,27]. This interpretation applies only once the finding has been shown to be isolated according to the criteria set out in the section titled “Operational Definition of an Isolated Finding”; until that assessment is complete, mild ventriculomegaly should be treated as a finding requiring structured evaluation rather than as a variant. Asymmetric VM is defined as an AD difference of >2 mm, with one or both ventricles > 10 mm [28]; unilateral dilation occurs in half of cases [25] but has similar outcomes to bilateral VM [29]. Image-quality challenges persist, attributable to maternal body habitus, respiratory motion, abdominal scarring, anterior placenta, oligohydramnios, non-cephalic presentation, and fetal movement [26].

All measurements described herein follow the ISUOG practice guidelines for the routine mid-trimester examination of the fetal brain: the atrial diameter is obtained in the axial transventricular plane with calipers placed inner-to-inner, perpendicular to the long axis of the ventricle, and any measurement ≥ 10 mm—or any suspicion of an associated anomaly—constitutes an indication for referral for dedicated fetal neurosonography [27].

### 3.4. Magnetic Resonance Imaging (MRI)

Fetal MRI provides excellent tissue contrast, superior soft-tissue resolution, a large field of view, and whole-fetus visualization [30], allowing improved detection of associated cerebral abnormalities through T2-weighted sequences, diffusion-weighted imaging, and tractography [31,32]. In selected cases, even ultra-high-field (7T and above) MRI may offer additional detail [33]. More recently, artificial intelligence-based reconstruction algorithms have been proposed to reduce motion-related artifacts; these tools are promising but remain investigational, not yet standardized across platforms or validated for routine clinical decision-making [34].

MRI demonstrates higher sensitivity than ultrasound, particularly after 24 weeks [35] (Figure 3). In the MERIDIAN study (570 cases), MRI in addition to ultrasound improved CNS-lesion detection from 82% to 95% [36].

### 3.5. Genetic Evaluation

Patients with fetal MV should be offered invasive genetic testing via amniocentesis. Although apparently isolated MV is associated with CMA abnormalities in only ~6.2% of cases, compared with 24.1% when additional malformations are present, genetic evaluation remains warranted [37]. CMA is currently the most widely used modality; WES/WGS are increasingly available as second-tier approaches (WES yield 8.5–10% with a normal CMA), though their utility in isolated MV remains to be established [38].

### 3.6. Prognosis and Counseling Considerations

In a cohort of 92 cases of isolated MV, Perlman et al. reported normal neurodevelopment in all infants except three, who had very mild deficits at 24 months [39]. Griffiths et al. reported favorable outcomes in approximately 90% of affected children [40]. Several caveats must be communicated: most prognostic studies have short follow-up (6–24 months), rely on unstandardized assessments rather than validated scales, and are subject to referral bias and loss to follow-up.

Parents should be informed that while the evidence supports a favorable prognosis in the majority of cases, ongoing neurodevelopmental surveillance through infancy and early childhood is advisable [40]. The comparative characteristics of the principal MV studies are summarized in Table 1 [10,15,37,39,40,41,42,43,44].

**Table 1:** Mild ventriculomegaly—comparative summary of studies.

Study Type & Source	n	Definition of “Isolated”	Imaging Confirmation	Neurodevelopmental Assessment (Tool)	Follow-Up Duration	Main Outcome
**Prospective cohort (imaging natural history)** Perlman et al., 2018 [39]	92	AD ≥ 10 mm with normal serology, anatomy scan, fetal MRI, genetic counseling and amniocentesis	US + fetal MRI; postnatal imaging	Not assessed (imaging focus) †	Postnatal (imaging) †	Significant postnatal regression of ventriculomegaly
**Prospective cohort** Sun et al., 2022 [41]	94	IVM ≥ 10 mm; mild 10–12 vs. moderate 12.1–15 mm; progression = ≥3 mm on serial US	Serial US	ASQ-3 and BSID-I	12 months	Outcomes compared by severity; mild subgroup more favourable
**Retrospective cohort** Gómez-Arriaga et al., 2023 [42]	43	Mild isolated VM 10–12 mm, isolated at diagnosis	US (±follow-up); 5/43 later structural abnormality	Battelle Developmental Inventory (BDI), 5 domains	Mid-term (children ~2–6 y)	Mostly favourable; 11% developed structural abnormalities on follow-up
**Retrospective cohort** Wang et al., 2024 [43]	324	Mild VM 10–12 mm (any GA); early- vs. late-onset	US	ASQ-3 + telephone interview	Postnatal (not to school age)	~11% neurodev delay (early 19.4% vs. late 7.4%); non-isolated status & progression predicted abnormality
**Cohort (in-utero MRI, MERIDIAN-linked)** Griffiths et al., 2022 [40]	NA	Isolated mild VM (IMVM) on MRI, no other abnormality	In-utero MRI	Not stated in abstract †	Not stated in abstract †	Very high chance of good outcome, esp. unilateral IMVM; outcome not linked to ventricular/brain volume
**Systematic review & meta-analysis** Ali et al., 2024 [44]	NA	IVM ≥ 10 mm from 15 wk, no additional finding	US ± MRI across studies	Heterogeneous (CAT/CLAMS, Griffiths, Amiel-Tison, BDIST, SDQ, Bayley)	1 month—14.6 years	Majority normal, esp. mild VM; documents wide heterogeneity in definitions, follow-up and tools
**Systematic review** Devaseelan et al., 2010 [15]	NA	Isolated mild-to-moderate VM	US ± MRI across studies	Heterogeneous †	Variable	Generally favourable; abnormal outcome more likely with progression or associated anomalies
**Retrospective cohort (genetics)** Toren et al., 2020 [37]	NR †	Isolated vs. additional malformations	US; CMA	Genetic (not neurodev)	Prenatal	Abnormal CMA 6.2% isolated vs. 24.1% with additional malformations
**Retrospective cohort** Ma’ayeh et al., 2021 [10]	NR †	Isolated VM that resolves in utero	Serial US	Clinical †	Postnatal	Favourable outcome when isolated VM resolves antenatally

AD, atrial diameter; ASQ-3, Ages & Stages Questionnaire 3rd ed.; BDI, Battelle Developmental Inventory; BSID-I, Bayley Scales of Infant Development I; CMA, chromosomal microarray; GA, gestational age; IMVM/IVM, isolated (mild) ventriculomegaly; MRI, magnetic resonance imaging; NA, not applicable; NR, not reported; US, ultrasound; VM, ventriculomegaly. † Not reported or not specified in the source publication.

## 4. Non-Dilated Ventricular Asymmetry (NDVA)

Functional and structural asymmetry of the brain is considered a normal developmental phenomenon [45], often side-dependent, with the left side larger than the right in normal fetuses and newborns [46,47,48]. It is more common in male fetuses (up to 70.8%) [1].

NDVA is defined as a difference of ≥2 mm between the atrial widths of the two lateral ventricles, both remaining below the threshold for ventriculomegaly (AD < 10 mm) [49,50] (Figure 1c). Asymmetry within the normal width range is generally considered benign [51]; however, Sgayer et al. report progression to ventriculomegaly in 5% to 76.4% of cases [52], a wide range reflecting heterogeneity in definitions, techniques, and small samples.

### 4.1. Etiology

Although the genetic risk is only slightly increased in MV cases, amniocentesis is preferred in most cases, even when the NIPT screening results are normal, as NIPT primarily targets numerical aneuploidies and cannot routinely detect copy number variants (microdeletions/microduplications) identified by chromosomal microarray (CMA) [1,52].

Only one study has associated NDVA with CMV infection, in a case with an intraventricular adhesion [53]; the paucity of data precludes definitive conclusions.

### 4.2. Ultrasound Assessment

Abdominal and especially transvaginal ultrasound are more accurate than MRI for measuring ventricular width, the main advantage of MRI being its ability to detect coexisting minor CNS anomalies (Figure 4). Measurements by MRI tend to be larger than those by ultrasound [1,54]. Ultrasound is sufficient for monitoring progression; where ventricular size does not exceed 15 mm, resolution before birth is likely [55].

In line with ISUOG guidance, both atria were considered adequately assessed only when measured in the standardized transventricular plane using the inner-to-inner caliper technique, as asymmetry values are highly sensitive to plane obliquity and caliper placement [51]. Targeted neurosonography is reserved for cases with an additional finding or measurement uncertainty [27].

### 4.3. Magnetic Resonance Imaging

Although MRI offers better characterization of CNS abnormalities, its added value in isolated NDVA is limited for the primary finding (Figure 5). However, Meyer et al. reported that MRI detects coexisting lesions not apparent on ultrasound [1], and Petpichetchian et al. identified grade I germinal matrix hemorrhage in 12 of 17 fetuses (71%) that were not seen on ultrasound [55].

### 4.4. Genetic Evaluation

Meyer et al. performed amniocentesis and karyotyping in 56 cases of NDVA, with CMA in more than half; three abnormal CMA findings and one chromosome 11 inversion were identified [1]. Sgayer et al. emphasize that the true rate of genetic abnormalities cannot be accurately assessed, and that it is unclear whether testing in the largest study was confined to isolated cases [52].

### 4.5. Prognosis and Counseling Considerations

In the absence of other CNS or genetic anomalies, prognosis appears favorable, though the evidence base is limited. Achiron et al. reported normal development at 6 months in 21 infants [53]; Sadan et al. reported similar findings in 21 cases [50], later re-examined by Atad-Rapoport et al. at 9–11 years, with most parameters not differing from controls [29]. Petpichetchian et al. followed 17 infants with normal results at 1 month, a period clearly insufficient to detect subtle delays [55].

Clinicians should communicate both the possibility of antenatal progression and the generally high likelihood of spontaneous resolution [52,55]. The comparative characteristics of the principal NDVA studies are summarized in Table 2 [1,29,49,50,52,53,55,56].

**Table 2:** Non-dilated ventricular asymmetry—comparative summary of studies.

Study Type & Source	n	Definition of “Isolated”	Imaging Confirmation	Neurodevelopmental Assessment (Tool)	Follow-Up Duration	Main Outcome
**Historical cohort** Meyer et al., 2021 [1]	145	Asymmetry ≥ 2 mm; both ventricles < 10 mm (dilation > 10 mm excluded)	US + fetal MRI; karyotype/CMA	Not assessed (genetics/imaging) †	Prenatal (genetic/imaging)	Abnormal karyotype 1.8% (1/56), CMA 10% (3/30); MRI added no major finding
**MRI-based cohort (vs. controls)** Meyer et al., 2018 [56]	43	IVA: asymmetry ≥ 2 mm, AD ≤ 10 mm, no VM	US → fetal MRI	Vineland-II (VABS-II)	13–74 months	Neurodev scores normal, no difference vs. 94 controls (109.6 vs. 107.8; *p* = 0.69)
**Systematic review** Sgayer et al., 2025 [52]	NA	Asymmetry ≥ 2 mm without dilation	US or MRI across studies	Heterogeneous; incl. school-age testing	First year to 9–11 years	36.8–46.2% progress to VM; genetic abnormality 5.1%; normal in year 1; at 9–11 y lower writing speed, higher verbal fluency
**Case series** Sadan et al., 2007 [50]	21	Asymmetric ventricles identified in utero (both <10 mm)	US (prenatal)	Bayley Scales of Infant Development II (BSID-II)	Infancy	Asymmetric group: normal mental/motor indices but higher risk of behavioural abnormalities vs. controls
**Case series (re-examination of the Sadan cohort)** Atad-Rapoport et al., 2015 [29]	21	Asymmetric ventricles/unilateral mild VM in utero	US (prenatal)	Neuropsychological battery (school age)	9–11 years	Most parameters no significant difference vs. controls
**Case series** Achiron et al., 1997 [53]	21	Cerebral lateral ventricular asymmetry on US	US (prenatal)	Clinical developmental assessment	6 months	Normal at 6 months; 20% resolved, 5% progressed, 75% stable
**MRI-based study** Barzilay et al., 2017 [49]	NR †	VM or asymmetry, MRI-based	Fetal MRI	Not assessed (imaging) †	Prenatal	Characterises additional brain anomalies associated with VM/asymmetry on MRI
**Retrospective case series** Petpichetchian et al., 2023 [55]	17	NDVA on US; both ventricles < 10 mm	US + fetal MRI	Neonatal exam + transfontanellar US (no long-term) †	1 month	Grade I germinal-matrix haemorrhage 12/17 (71%) on MRI, not on US; normal at 1 month

AD, atrial diameter; BSID-II, Bayley Scales of Infant Development II; CMA, chromosomal microarray; CNV, copy-number variation; CNS, central nervous system; IVA, isolated ventricular asymmetry; MRI, magnetic resonance imaging; NA, not applicable; NDVA, non-dilated ventricular asymmetry; NR, not reported; US, ultrasound; VABS-II, Vineland Adaptive Behavior Scales II; † Not reported or not specified in the source publication.

## 5. Mega Cisterna Magna (MCM)

MCM is classically defined as a cystic enlargement of the posterior fossa in the absence of hydrocephalus, with an intact cerebellar vermis, arising embryologically from the permeabilization of Blake’s pouch (Figure 6) [57,58]. It occurs more frequently in male fetuses [59]. The incidence of isolated MCM is relatively low, ranging from 0.25 to 0.33% [60,61].

### 5.1. Embryology Review and Anatomic Landmarks

Blake’s pouch is an inferior protrusion of the fourth ventricle, which later becomes permeable and constitutes the foramen of Magendie [62]. The cisterna magna extends anteriorly to the foramen of Magendie and inferiorly to the first cervical level [63]. The foramen of Magendie is a key midline sagittal landmark, since its patency differentiates a normal posterior fossa from Blake’s pouch cyst.

### 5.2. Etiology

Some studies associate MCM with intrauterine viral infections (CMV), which may obstruct the fenestrations of Luschka and Blake’s pouch; however, such cases typically present additional CNS abnormalities [64]. Isolated MCM without associated findings is unlikely to be of infectious origin.

### 5.3. Ultrasound Assessment

Standard evaluation involves assessing the anteroposterior diameter on the transcerebellar section [51] (Figure 7a,b); MCM is suspected when it exceeds 10 mm on mid-sagittal images [65] (Figure 7c). Wuest et al. diagnosed 29 cases by antenatal ultrasound, of which 6 (21%) had additional CNS abnormalities [7].

Following ISUOG recommendations, the cisterna magna is measured in the transcerebellar plane between the vermis and the inner border of the occipital bone, with values >10 mm prompting referral for detailed neurosonographic assessment of the posterior fossa [27,51].

The measurement technique deserves emphasis because the cisterna magna is particularly vulnerable to plane-related error: an oblique transcerebellar section passing through the inferior vermis, or a semi-coronal plane obtained in a breech or deeply engaged fetus, artefactually elongates the anteroposterior diameter and may create the appearance of MCM where none exists. The measurement should therefore be taken in a plane displaying the cavum septi pellucidi, both cerebellar hemispheres and the vermis simultaneously, with the calipers placed from the posterior border of the vermis to the inner table of the occipital bone. Whenever the depth approaches or exceeds 10 mm, the finding should be confirmed in the midsagittal plane—ideally transvaginally in cephalic presentation—where an intact vermis, a normally positioned fastigium and free communication of the fluid space with the fourth ventricle distinguish MCM from Blake’s pouch cyst, arachnoid cyst and the Dandy–Walker continuum [27,51].

### 5.4. Magnetic Resonance Imaging

Fetal MRI offers superior soft-tissue differentiation when ultrasound cannot accurately characterize the posterior fossa or distinguish it from DWC, detecting previously unidentified secondary anomalies in up to 60% of cases [66,67]. The general recommendation is that neurosonography be complemented by MRI [68] (Figure 8).

### 5.5. Genetic Evaluation

Discrepancies regarding the risk of aneuploidy in MCM result from inconsistent reporting of isolated versus associated cases. Wuest et al. found an abnormal karyotype in 4/13 overall, but all truly isolated cases were euploid [7]. Lei et al. found no aneuploidies in isolated MCM or MCM with only other CNS abnormalities, but identified 2 cases of trisomy 18 and 1 case of trisomy 21 in the extra-CNS group [69].

### 5.6. Prognosis and Counseling Considerations

The neurological prognosis for isolated MCM is generally favorable [59,70,71,72,73]. Some childhood and adult studies suggest possible associations with neurodevelopmental delay and psychiatric disorders [74,75,76,77,78,79,80,81,82,83], but causality is uncertain. D’Antonio et al. reported that isolated MCM is generally favorable, with possible intrauterine regression [84]; Kim et al. confirmed self-regression in two-thirds of cases [59].

If the condition persists after birth, a small proportion of children may show developmental delay, underlining the need for prenatal and postnatal evaluation [84]. The comparative characteristics of the principal MCM studies are summarized in Table 3 [7,59,60,67,68,69,77,78,83,84].

**Table 3:** Mega cisterna magna—comparative summary of studies.

Study Type & Source	n	Definition of “Isolated”	Imaging Confirmation	Neurodevelopmental Assessment (Tool)	Follow-Up Duration	Main Outcome
**Multicenter retrospective cohort** Kim et al., 2024 [59]	71	CM > 10 mm, normal vermis, no 4th-ventricle cystic dilation; CNS, extra-CNS, chromosomal and infection causes excluded	US ± MRI; postnatal	Clinical follow-up (tool NR) †	Prenatal–postnatal	Self-regression 48/71 (67.6%); persistent 13 (18.3%); 10 (14.1%) other CNS anomaly; 1 child delayed
**Systematic review & meta-analysis** D’Antonio et al., 2016 (part 2) [84]	NA	No associated cerebral/extracerebral anomaly at diagnosis	US ± MRI across studies	Heterogeneous across studies	Variable	Isolated MCM favourable; may regress; small proportion with delay if persistent
**Retrospective case series** Wüest et al., 2017 [7]	29	Enlarged posterior fossa; isolated vs. associated	US + MRI; postnatal/autopsy	Clinical (postnatal) †	Prenatal–postnatal	6/29 (21%) additional CNS anomaly; karyotype abnormal 4/13 overall, isolated cases euploid
**Retrospective cohort (genetics)** Lei et al., 2017 [69]	NR †	Isolated MCM vs. MCM with CNS vs. extra-CNS anomalies	US; karyotype/CNV	Genetic (not neurodev)	Prenatal	No aneuploidy in isolated MCM or MCM+CNS; T18 ×2 and T21 ×1 only with extra-CNS anomalies
**Observational study** Zimmer et al., 2007 [60]	NR †	Isolated MCM	US	Clinical †	Postnatal	Addresses clinical significance of isolated MCM; generally benign
**Case series** Dror et al., 2009 [77]	NA	Enlarged cisterna magna in utero	US (prenatal)	Neuropsychological testing	Childhood	Isolated cases scored lower on memory, executive function and verbal fluency vs. controls
**Case series** Gandolfi Colleoni et al., 2012 [78]	NR †	Posterior fossa fluid collections; isolated vs. associated	US + MRI	Clinical follow-up †	Postnatal	Neurodevelopmental delay in up to ~20% when additional anomalies present
**Retrospective cohort** Şeker et al., 2023 [68]	NR †	Posterior fossa anomalies on fetal MRI	Fetal MRI	Clinical/neurodev follow-up †	Long-term	Long-term outcomes of posterior fossa anomalies diagnosed by fetal MRI
**Case series (preliminary, single-centre)** Pishjoo et al., 2025 [67]	NR †	Isolated MCM	US ± MRI	Clinical follow-up †	Postnatal	Preliminary data; generally favourable outcome reported
**Case report (adult)** Yazici et al., 2022 [83]	1	Radiological MCM (>10 mm), incidental	CT/MRI (postnatal)	Psychiatric diagnosis (adult)	Adult	MCM incidentally associated with bipolar mood disorder—a possible, unproven association

CM, cisterna magna; CNV, copy-number variation; CNS, central nervous system; DWM, Dandy–Walker malformation; MCM, mega cisterna magna; MRI, magnetic resonance imaging; NA, not applicable; NR, not reported; US, ultrasound; † Not reported or not specified in the source publication.

## 6. Blake’s Pouch Cyst (BPC)

Blake’s pouch cyst (BPC), also termed persistent Blake’s pouch, is considered a rare posterior fossa anomaly, with a reported incidence of approximately 1 per 1000 pregnancies [62]. In contrast to MCM, persistent Blake’s pouch can be associated with hydrocephalus, but is not associated with cerebellar hypoplasia or other brain abnormalities (Figure 9).

### 6.1. Etiology

BPC represents an outpouching of the fourth ventricle into the cisterna magna, caused by failure of fenestration of the posterior membranous area [62]. In contrast to Dandy–Walker malformation—in which the vermis is hypoplastic, the brainstem–vermis angle exceeds 45°, and the posterior fossa is enlarged—BPC preserves vermian morphology and posterior fossa dimensions [85].

### 6.2. Ultrasound Assessment

Typical findings include a normally sized vermis, mildly to moderately rotated antero-superiorly on sagittal section, with a normal cisterna magna (Figure 10a). A “keyhole” appearance may be seen when the cyst is large (Figure 10b) [86].

Obtaining a true midsagittal plane is the key technical step: transabdominally, it is acquired through the sagittal suture or the anterior fontanelle, and in cephalic presentation, the transvaginal route offers superior resolution of the vermis, the fastigium and the cyst walls. Slight parasagittal or oblique planes foreshorten the vermis and may either mimic or conceal its upward rotation, so the plane should be validated by the simultaneous display of the brainstem, fourth ventricle and vermis before any angle is measured [27].

According to the Society for Maternal-Fetal Medicine, the brainstem–vermis angle is a helpful discriminator: it is close to 0° in the normal posterior fossa, usually 18–45° in Blake’s pouch cyst, and 45° or greater in Dandy–Walker malformation [7,86] (Figure 10c). The brainstem–vermis angle carries much of the diagnostic weight in this differential, and its limitations should be acknowledged. The measurement requires a true midsagittal plane, and small deviations in plane, in the placement of the brainstem line along the dorsal surface, or in the identification of the anterior vermian border shift the angle by several degrees. The reported reference ranges reflect this: the 5–45° range quoted for Blake’s pouch cyst abuts the normal range at its lower end and the Dandy–Walker range at its upper end, so that values in the region of 40–50° do not reliably separate the two entities. Where the angle falls in this equivocal range, classification should rest on the additional discriminating features—vermian size and lobulation, the position of the fastigium, the dimensions of the posterior fossa and the presence of tentorial elevation—and on serial assessment, rather than on the angle alone [27,51].

In a meta-analysis of 86 fetuses with a prenatal diagnosis of BPC, the rate of associated anomalies was 11.5% for CNS and 25.3% for extra-CNS anomalies [85].

As emphasized in the ISUOG neurosonography guideline, the distinction between Blake’s pouch cyst and other cystic posterior fossa anomalies requires dedicated multiplanar neurosonography—ideally transvaginal, in the midsagittal plane—to assess vermian integrity and the position of the fastigium, rather than relying on the axial screening planes alone [27].

### 6.3. Magnetic Resonance Imaging

On MRI, BPC is best characterized on midline sagittal T2-weighted sequences as a thin-walled, hyperintense cystic structure communicating with a dilated fourth ventricle, with an intact but upward-rotated vermis (brainstem–vermis angle 18–45°) and normal posterior fossa (Figure 11) [78,87,88]. In a meta-analysis including 45 fetuses with isolated BPC, MRI identified no additional CNS anomaly, and MRI and ultrasound were equivalent for vermis and angle measurement [85].

A 2025 study highlights a potential benefit of MRI over ultrasound in differentiating BPC from posterior fossa lesions with a worse prognosis, through more accurate measurement of vermis size and the brainstem–vermis angle [89].

### 6.4. Genetic Evaluation

Isolated BPC is rarely associated with chromosomal abnormalities [84,85]. Guo et al. reported an overall aberration rate of 31.8% (7/22); however, only 1 case of trisomy 21 was found in isolated BPC, with the remainder having other structural abnormalities [90]. The SMFM advises karyotype and CNV analysis when BPC is detected, especially for non-isolated BPC, with cell-free DNA offered when invasive testing is declined [86].

### 6.5. Prognosis and Counseling Considerations

One third to 50% of isolated BPC cases may resolve in utero during the third trimester [72,91,92]. In 111 cases, Salsi et al. concluded that isolated BPC with upward vermian rotation may best be described as a normal variant [92]. Normal neurodevelopmental outcomes occur in approximately 95–100% of isolated cases [72,84,85,91]. With additional CNS or extra-CNS malformations, especially with genetic abnormalities, neurodevelopmental delay may be observed in up to 20% of cases [78].

Parents should be counseled that while isolated BPC carries an excellent prognosis, postnatal surveillance for hydrocephalus is warranted, and additional anomalies significantly alter the outlook. The comparative characteristics of the principal BPC studies are summarized in Table 4 [72,73,78,85,89,90,91,92,93].

**Table 4:** Blake’s pouch cyst—comparative summary of studies.

Study Type & Source	n	Definition of “Isolated”	Imaging Confirmation	Neurodevelopmental Assessment (Tool)	Follow-Up Duration	Main Outcome
**Retrospective cohort** Salsi et al., 2021 [92]	111	Isolated upward rotation of vermis (BPC); normal posterior fossa	US ± MRI	Clinical follow-up (tool NR) †	Prenatal–postnatal	Isolated BPC behaves as a normal variant
**Systematic review & meta-analysis** D’Antonio et al., 2016 (part 1) [85]	NA	No associated anomaly at diagnosis	US + MRI across studies	— (part 1: diagnostic accuracy/anomalies)	—	MRI added no CNS anomaly in 45 isolated cases; associated anomalies (n = 86): CNS 11.5%, extra-CNS 25.3%
**Retrospective case series** Behram et al., 2021 [91]	19	BPC on prenatal imaging; isolated vs. associated	US ± MRI	Clinical (postnatal) †	Prenatal–postnatal	Isolated 9/19 (47%); associated 10/19 (53%): 7 CNS, 4 cardiac
**Case series (CNV analysis)** Guo et al., 2024 [90]	22	Prenatally diagnosed BPC; isolated vs. non-isolated	US ± MRI; CMA/CNV	Not assessed (genetics focus) †	Prenatal	Chromosomal aberration 31.8% (7/22); only 1 isolated case (trisomy 21); rest had other anomalies
**Case series/natural history** Paladini et al., 2012 [72]	11	Isolated BPC (upward vermian rotation)	US (±MRI)	Clinical follow-up †	Prenatal–postnatal	Regression in 6/11 (55%) by term; vermis normalised by 24–26 weeks
**Case series** Gandolfi Colleoni et al., 2012 [78]	NR †	Posterior fossa fluid collections; isolated vs. associated	US + MRI	Clinical follow-up †	Postnatal	Neurodevelopmental delay in up to ~20% when additional anomalies present
**Case series (fetal MRI)** Patek et al., 2012 [73]	NR †	Posterior fossa anomalies on fetal MRI	Fetal MRI	Neurodevelopmental follow-up †	Postnatal	Associated anomalies and neurodevelopmental outcomes of posterior fossa anomalies on MRI
**Biometric study (fetal MRI)** Bowker et al., 2025 [89]	NR †	Dandy–Walker-spectrum posterior fossa lesions	Fetal MRI biometry	— (diagnostic accuracy)	—	MRI biometry improves antenatal differentiation of BPC from DWM/vermian hypoplasia
**Case report** Azab et al., 2014 [93]	1	Persistent BPC with hydrocephalus	MRI (postnatal)	Clinical/neurosurgical	Postnatal	Symptomatic hydrocephalus treated with endoscopic ventriculostomy

CMA, chromosomal microarray; CNV, copy-number variation; CNS, central nervous system; DWM, Dandy–Walker malformation; MRI, magnetic resonance imaging; NA, not applicable; NR, not reported; US, ultrasound; † Not reported or not specified in the source publication.

## 7. Integrated Discussion: Common Themes and a Framework for Clinical Practice

Several common themes emerge from the review of these four entities that warrant an integrated discussion rather than the compartmentalized approach often seen in the literature. MV, NDVA, MCM, and BPC should be viewed as points along a spectrum from normal anatomical variation to mild pathology; where a given finding falls depends not on the finding in isolation, as defined in the section titled “Operational Definition of an Isolated Finding”, but on the presence or absence of associated anomalies, the genetic background, and the longitudinal trajectory.

A further consideration cutting across all four entities is that these borderline findings are as liable to under-diagnosis as to over-diagnosis, and both errors carry real consequences. Under-recognition may allow a finding that is in fact the first sign of a progressive or syndromic process to go unmonitored, forfeiting the opportunity for timely reassessment, genetic testing or delivery planning. Over-diagnosis—labeling a normal variant as pathology or over-stating its implications—generates substantial and often lasting parental distress, sets off a cascade of unnecessary investigations, referrals and repeat scans, and consumes finite healthcare resources. Because the margin between normal variant and mild pathology is narrow and measurement-dependent, we would argue that the most effective safeguard against both is sustained investment in sonographer and physician training and in the standardization of diagnostic criteria, measurement techniques and reporting across centers.

The role of MRI varies across entities: for MV and MCM, it offers clear additive value by detecting associated anomalies, particularly after 24 weeks; for NDVA, its value is more nuanced; and for BPC, the evidence is mixed. Genetic testing requires a tiered approach, with CMA as the first-tier invasive test, WES/WGS as the second-tier when CMA is non-diagnostic, and NIPT as a screening alternative; invasive testing is more clearly justified in MV and non-isolated posterior fossa anomalies than in truly isolated NDVA, MCM, or BPC. These cross-entity patterns are summarized in Table 5, Table 6 and Table 7.

The corollary matters as much for counseling. There are contexts in which fetal MRI predictably adds little: an isolated finding assessed on high-quality multiplanar neurosonography by an experienced operator, in a fetus with a favorable position and adequate acoustic windows. In isolated non-dilated ventricular asymmetry and in isolated Blake’s pouch cyst, MRI has repeatedly been shown not to alter the diagnosis or the management plan [1,85]. MRI performed before 24 weeks is also less informative, since much of its advantage over ultrasound derives from the depiction of cortical development and of the posterior fossa in the third trimester. Set against the cost of the examination, the limited availability of fetal MRI outside tertiary centers, and the parental anxiety generated by a further test, indiscriminate use in these settings constitutes over-utilization rather than thoroughness. We would restrict fetal MRI to the indications set out in Table 6.

Contemporary prenatal evaluation increasingly integrates genomic testing with advanced imaging: CMA, and where indicated whole-exome or whole-genome sequencing, are interpreted alongside detailed neurosonography and fetal MRI [38]. The greatest value comes from multidisciplinary discussion involving maternal–fetal medicine, fetal neurology, clinical genetics and radiology.

The prognostic literature for all four entities shares common methodological limitations—small samples, heterogeneous definitions, short follow-up, unstandardized assessments, and referral and reporting biases—which clinicians should explicitly communicate. Favorable outcome rates should be conveyed alongside these caveats rather than as definitive reassurance, as the per-entity data in Table 1, Table 2, Table 3 and Table 4 make clear.

**Table 5:** Cross-entity overview of the four borderline findings.

Entity	Definition/Threshold	Prevalence	Main Differential	Genetic Yield If Isolated	Prognosis If Isolated
**MV**	AD 10–12 mm (mild)	~1–2/1000	Progressive VM; ACC; aqueductal stenosis	~6% CMA	~90% favourable; surveillance advised
**NDVA**	Asymmetry ≥ 2 mm; both <10 mm	Common variant	True VM; pseudo-asymmetry	~1.8% karyotype/~10% CMA (small n)	Favourable; frequent resolution
**MCM**	CM AP > 10 mm; intact vermis	~0.25–0.33%	BPC; DWC; arachnoid cyst	Rare if truly isolated	Favourable; ~two-thirds regress
**BPC**	Upward-rotated vermis; angle ~5–45°	~1/1000	MCM; DWM; vermian hypoplasia	<5% if isolated	Excellent (95–100%); may resolve

ACC, agenesis of the corpus callosum; AD, atrial diameter; CM, cisterna magna; CMA, chromosomal microarray; DWC/DWM, Dandy–Walker continuum/malformation; VM, ventriculomegaly.

**Table 6:** Comparative role of ultrasound and fetal MRI, by entity.

Entity	Primary Role of Ultrasound	Added Value of Fetal MRI	When MRI Changes Management	When MRI Adds Little
**MV**	Detect and measure AD; serial monitoring	Higher sensitivity for associated CNS anomalies, esp. > 24 wk (MERIDIAN 82 → 95%)	Equivocal ultrasound or suspected associated anomaly	Isolated MV of 10–12 mm with normal, high-quality multiplanar neurosonography; examination before ~24 wk, when cortical assessment is still immature
**NDVA**	Accurate ventricular width; monitor progression	Detects coexisting lesions (e.g., germinal-matrix haemorrhage) not seen on US	Progressive asymmetry or etiological counseling	Isolated NDVA—repeatedly shown not to alter diagnosis or management; ventricular measurements also read systematically larger on MRI
**MCM**	AP diameter; transcerebellar view	Differentiates from DWC; secondary anomalies up to 60%	Ambiguous posterior fossa or DWC differential	Isolated MCM with an intact vermis confirmed on good-quality (ideally transvaginal) midsagittal imaging
**BPC**	Vermis rotation; keyhole sign; angle	Often equivalent; helps exclude DWM/vermian hypoplasia	Equivocal cases or worse-prognosis differential	Isolated BPC with typical midsagittal features and normal vermian biometry—MRI usually confirms rather than changes the ultrasound diagnosis

AP, anteroposterior; DWC/DWM, Dandy–Walker continuum/malformation; US, ultrasound.

**Table 7:** Genetic evaluation and diagnostic yield, by entity and isolation status.

Entity	Yield If Isolated	Yield If Non-Isolated	Recommended Invasive Test	NIPT/cfDNA Role	Strength of Recommendation
**MV**	~6.2% CMA	~24.1% CMA	Amniocentesis + CMA (offer)	Screening alternative if invasive declined	Offer routinely (amniocentesis + CMA), including when apparently isolated
**NDVA**	~1.8% karyotype; ~10% CMA (small n)	Higher	Consider amniocentesis + CMA	NIPT if adequate screening	Discuss; offer where NIPT screening has not been performed or is inadequate
**MCM**	Rare/none if truly isolated	T18, T21 with extra-CNS anomalies	Generally only if associated anomalies	cfDNA if invasive declined	Not routinely indicated; offer if the family requests it after counselling
**BPC**	<5% if isolated	Up to ~32% overall (mostly non-isolated)	Karyotype + CNV, esp. non-isolated (SMFM)	cfDNA if invasive declined	Not routinely indicated; recommend if any additional anomaly is present

cfDNA, cell-free DNA; CMA, chromosomal microarray; CNV, copy-number variation; NIPT, non-invasive prenatal testing; T, trisomy.

A staged framework applicable to all four entities should be applied to rule out associated anomalies, provide adjunct methods for refining diagnosis, and counsel the couple (Figure 12). This framework is offered as a pragmatic, expert-opinion approach rather than a prospectively validated instrument; it has not been tested against neurodevelopmental endpoints, and its categories should be adapted to local resources and the individual clinical context, with distinct pathways for isolated and non-isolated findings.

The differential diagnosis is a recurring challenge: each entity is defined as much by what it is not as by what it is. Mild ventriculomegaly must be distinguished from progressive ventriculomegaly and from dilatation secondary to a structural or destructive lesion; NDVA from true ventriculomegaly and technical pseudo-asymmetry; and MCM and BPC from each other and from the Dandy–Walker continuum and arachnoid cysts. In each case, the distinction rests on a systematic assessment of a small number of discriminating features, and the correct classification carries substantial prognostic and counseling weight.

## 8. Conclusions and Future Directions

Sonographic evaluation remains the primary imaging modality in prenatal care due to its wide availability, low cost, repeatability, and ease of use. However, ultrasound has well-recognized limitations related to fetal position, maternal habitus, placental location, oligohydramnios, equipment quality, and examiner experience; certain associated abnormalities may require fetal MRI. It should also be borne in mind that the ultrasound system itself—its resolution and overall performance—may influence how reliably these subtle findings are detected. Borderline CNS findings are therefore best assessed on proficient equipment by experienced operators.

In cases of minor anomalies of the lateral ventricular system, amniocentesis and prenatal genetic testing should be offered because, even in apparently isolated cases, the risk of clinically significant genetic abnormalities—while relatively low—is not negligible. In contrast, isolated posterior fossa anomalies (MCM and BPC) are often considered anatomical variants when truly isolated, and prenatal genetic diagnosis is generally recommended only when additional abnormalities are identified.

Postnatal neurodevelopmental outcomes are generally favorable for all the discussed entities, provided they are not associated with underlying genetic or infectious conditions. However, the evidence base has important limitations that must be acknowledged, including the absence of a formal risk-of-bias appraisal and of a PRISMA flow diagram, as set out in Section 2.

Priorities for future research can be stated specifically. First, the field needs harmonized definitions—of “isolated” status and of the threshold measurements for each entity—so that cohorts become comparable. Second, prospective multicenter studies with standardized recruitment are required to overcome the small samples and referral bias. Third, neurodevelopmental follow-up should use validated batteries and extend to school age, when cognitive, language, and behavioral difficulties undetectable in infancy may first appear. Finally, advanced neuroimaging should be integrated with genomic analysis so that imaging phenotype and molecular findings can be interpreted together.

Until such data become available, counseling should be honest, balanced, and transparent about the limitations of current evidence. Parents should be reassured that the available data are broadly favorable, while being informed that ongoing postnatal surveillance is recommended and that the long-term evidence base—though growing—remains incomplete.

## Figures and Tables

**Figure 1:**
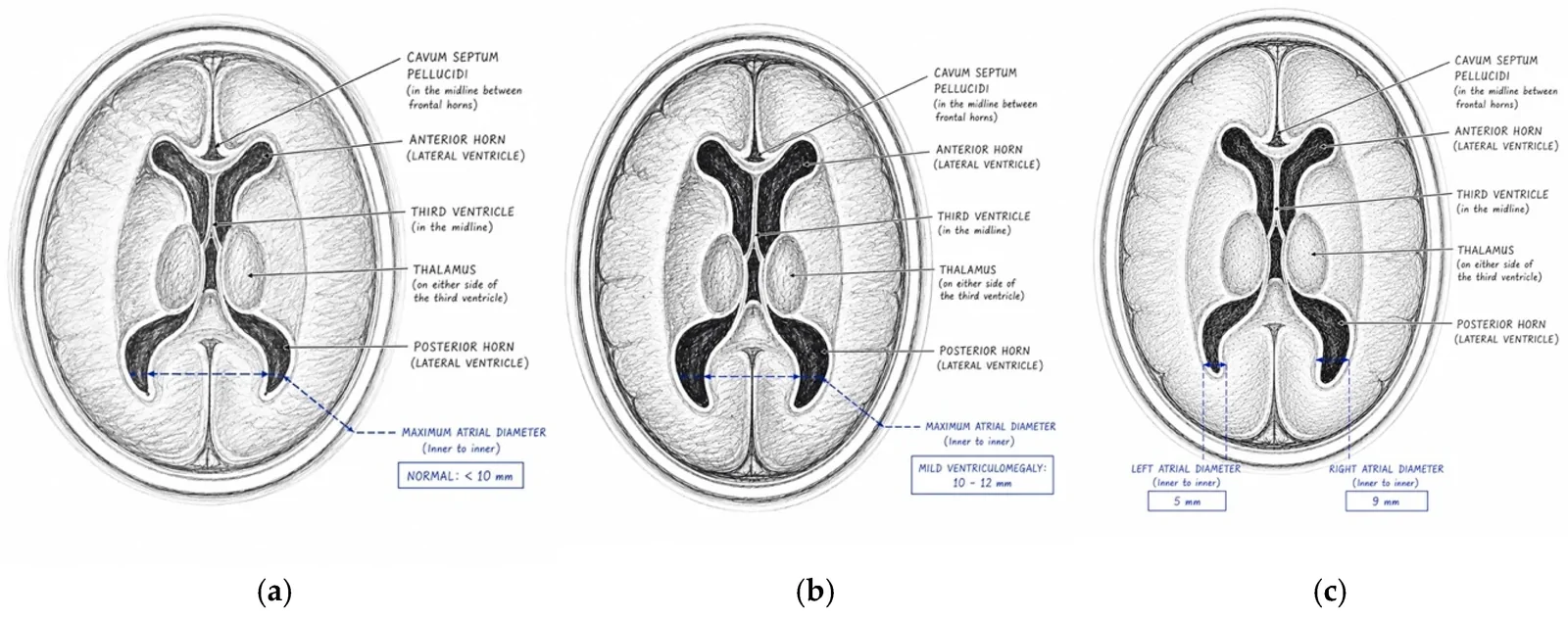
(**a**) Sketch of anatomic landmarks of fetal CNS axial scan plan; (**b**) sketch of mild ventriculomegaly (MV); (**c**) sketch of non-dilated ventricular asymmetry (NDVA).

**Figure 2:**
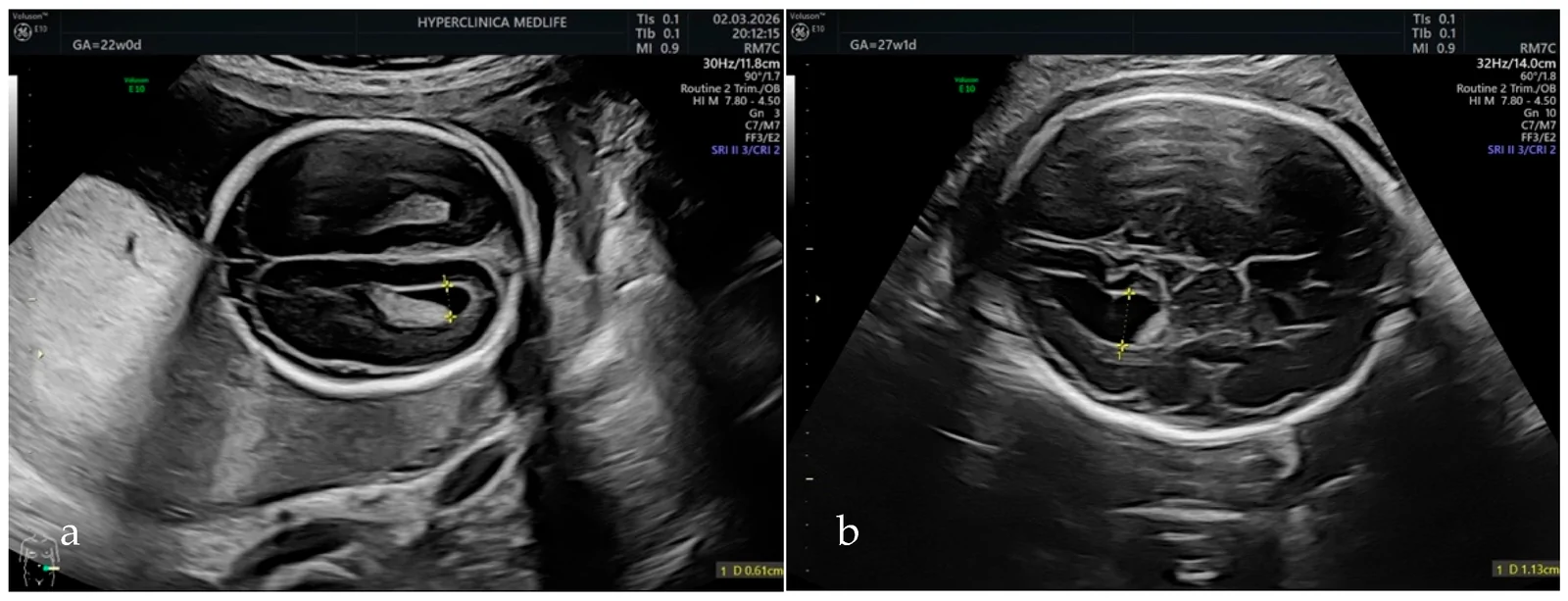
(**a**) Normal measurement of lateral ventricles in the axial transventricular plane. (**b**) Mild ventriculomegaly (AD = 11 mm). Both images obtained at 22 weeks of gestation.

**Figure 3:**
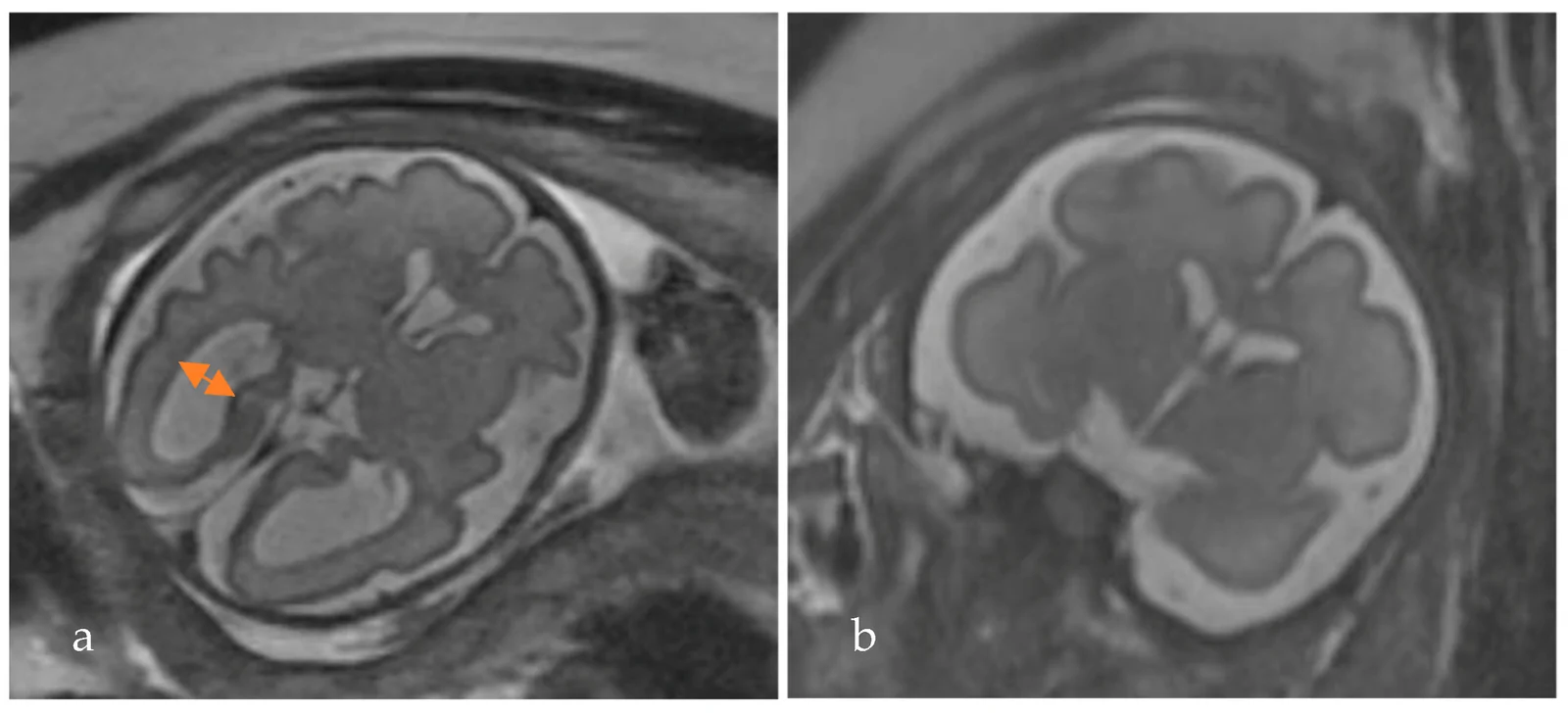
Fetal MRI at 29 weeks of gestation showing mild ventriculomegaly of 11 mm (orange arrow) (**a**), without other obvious associated lesions (**b**).

**Figure 4:**
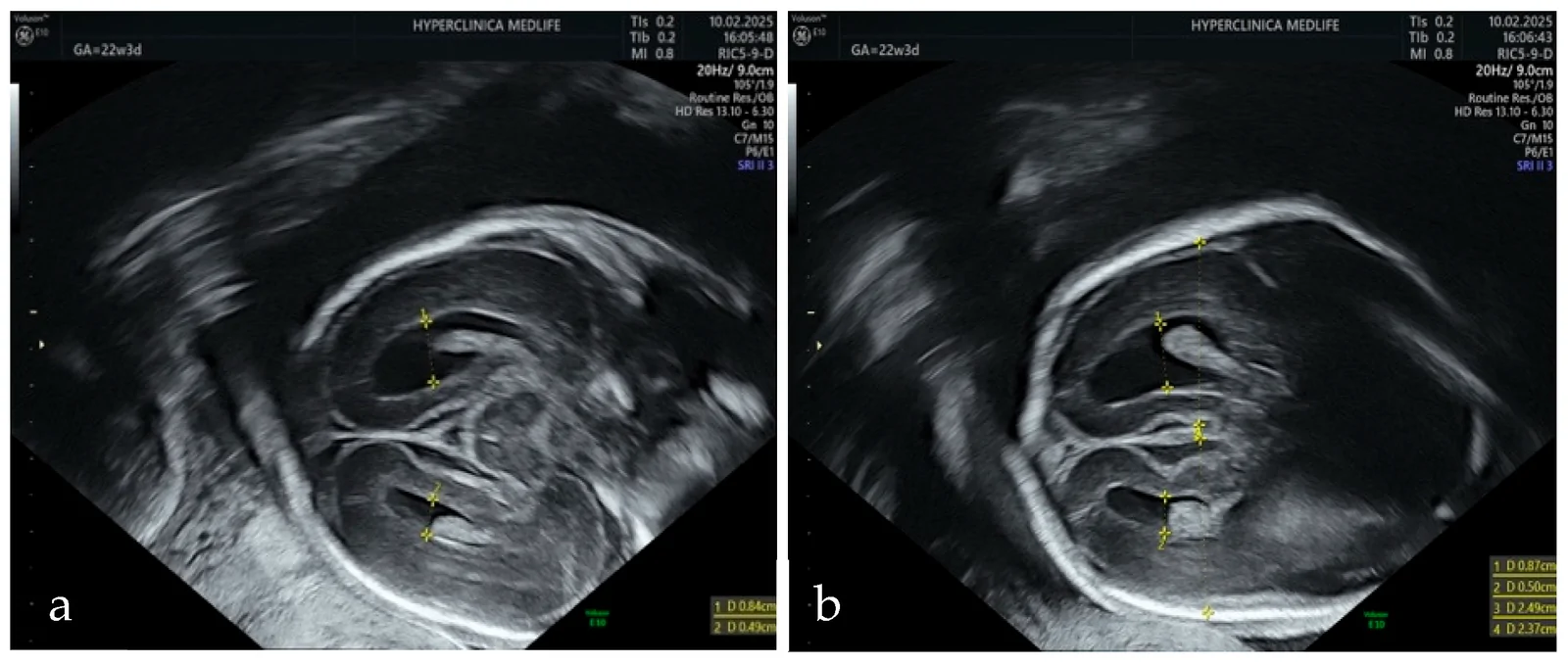
Fetal brain ventricular asymmetry on ultrasound at 22 (**a**,**b**) weeks of gestation (calipers).

**Figure 5:**
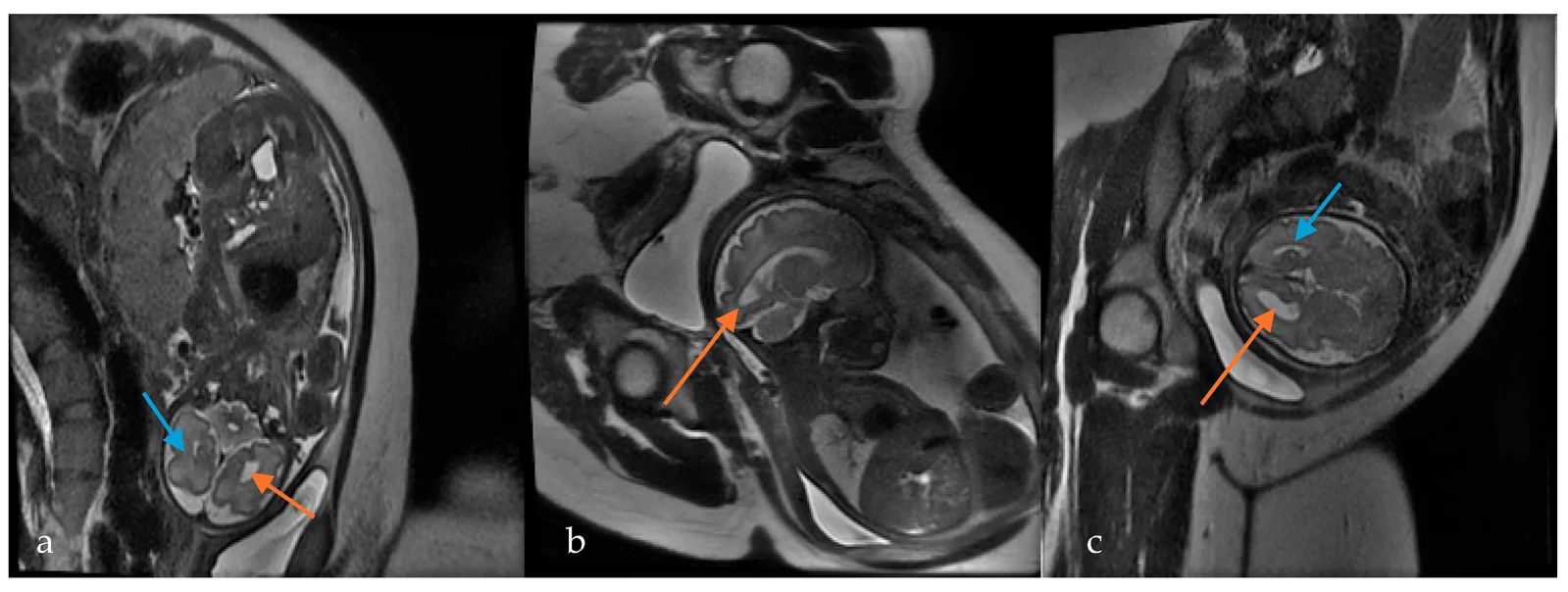
Fetal brain ventricular asymmetry on MRI in the same fetus as Figure 4, at 30 weeks of gestation. (**a**) Smaller ventricle (blue arrow, 4 mm). (**b**) Larger ventricle (orange arrow, 8.5 mm). (**c**) Lateral ventricle asymmetry on the axial plane. Note the systematically larger measurements on MRI compared to ultrasound (see Section 3.2).

**Figure 6:**
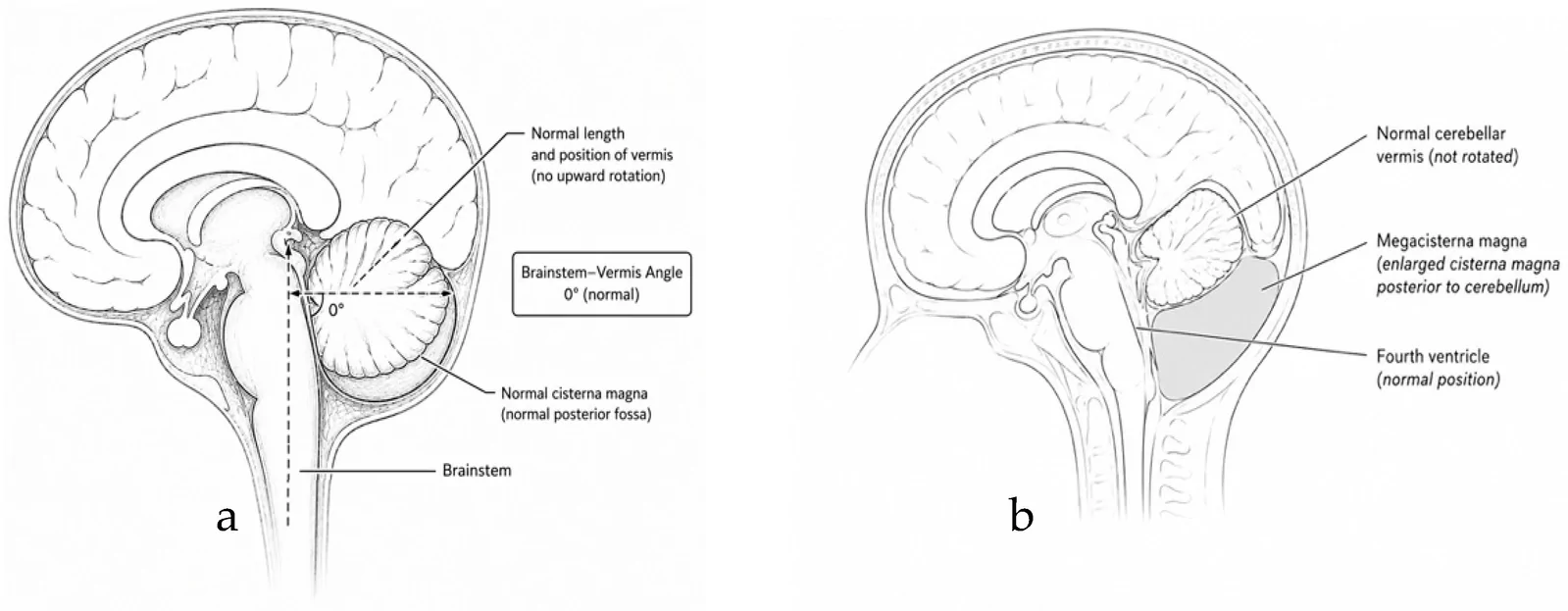
Sketch of the normal aspect of (**a**) cysterna magna and (**b**) megacisterna magna.

**Figure 7:**
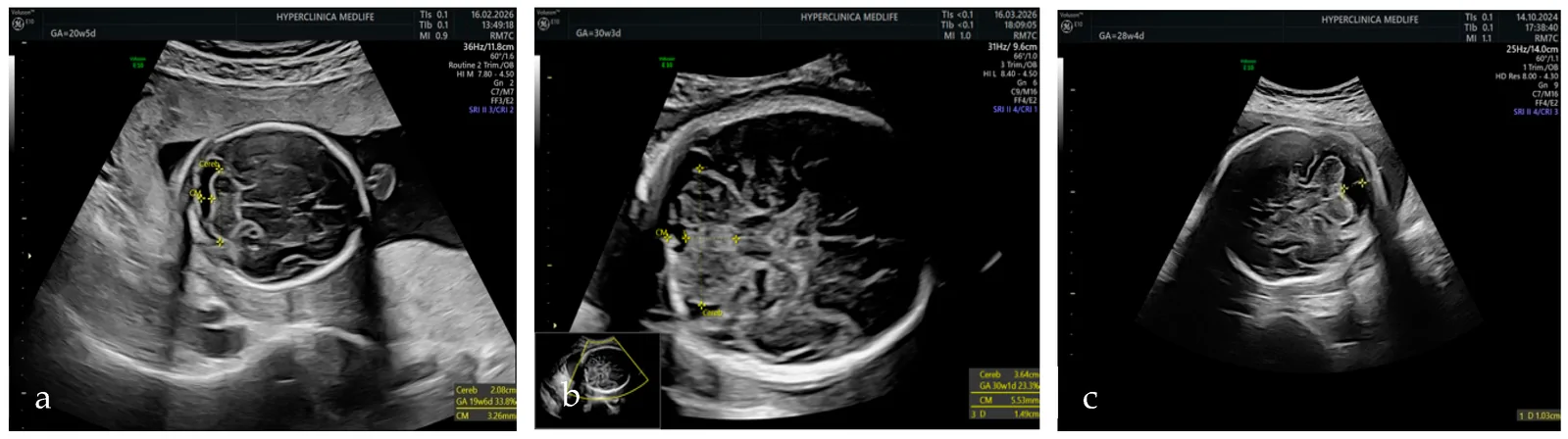
(**a**,**b**) Transcerebellar section (**c**) MCM at 28 WG.

**Figure 8:**
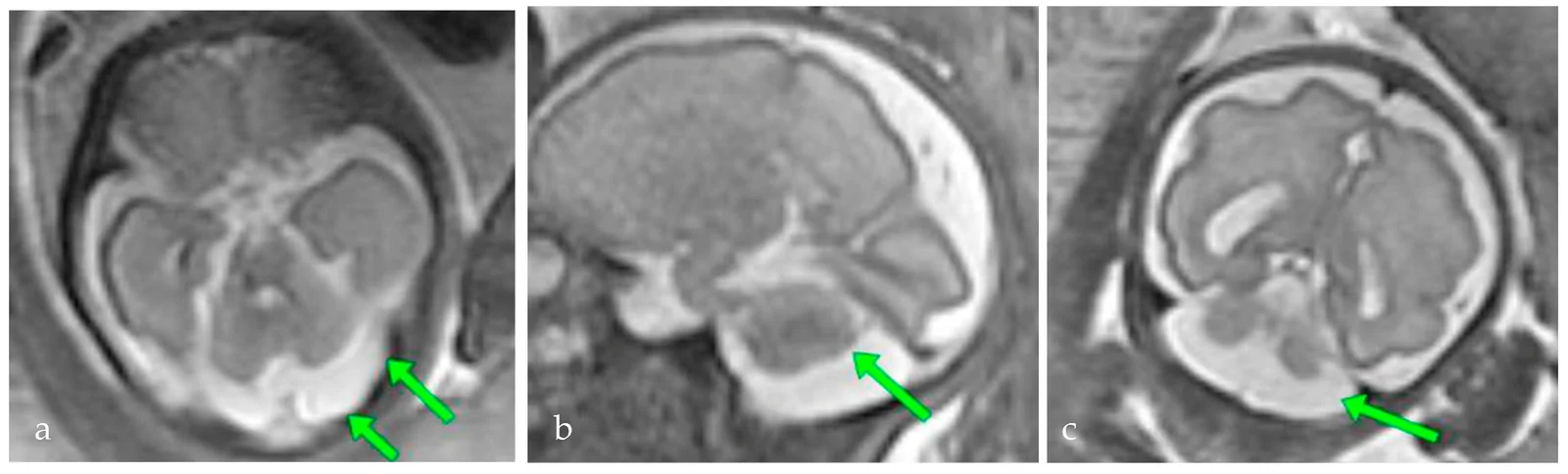
Fetal MRI demonstrating MCM at 30 weeks of gestation. (**a**) T2 axial sections at the level of the posterior fossa, with slight posterior cystic enlargement and minimal imprinting of the left cerebellar hemisphere (green arrow); (**b**) sagittal T2 sections at the level of the left cerebellar hemisphere (slightly imprinted), vermis and right cerebellar hemisphere (green arrow); (**c**) coronal sections with slight asymmetric widening of the cisterna magna and normal-appearing cerebellum (green arrow).

**Figure 9:**
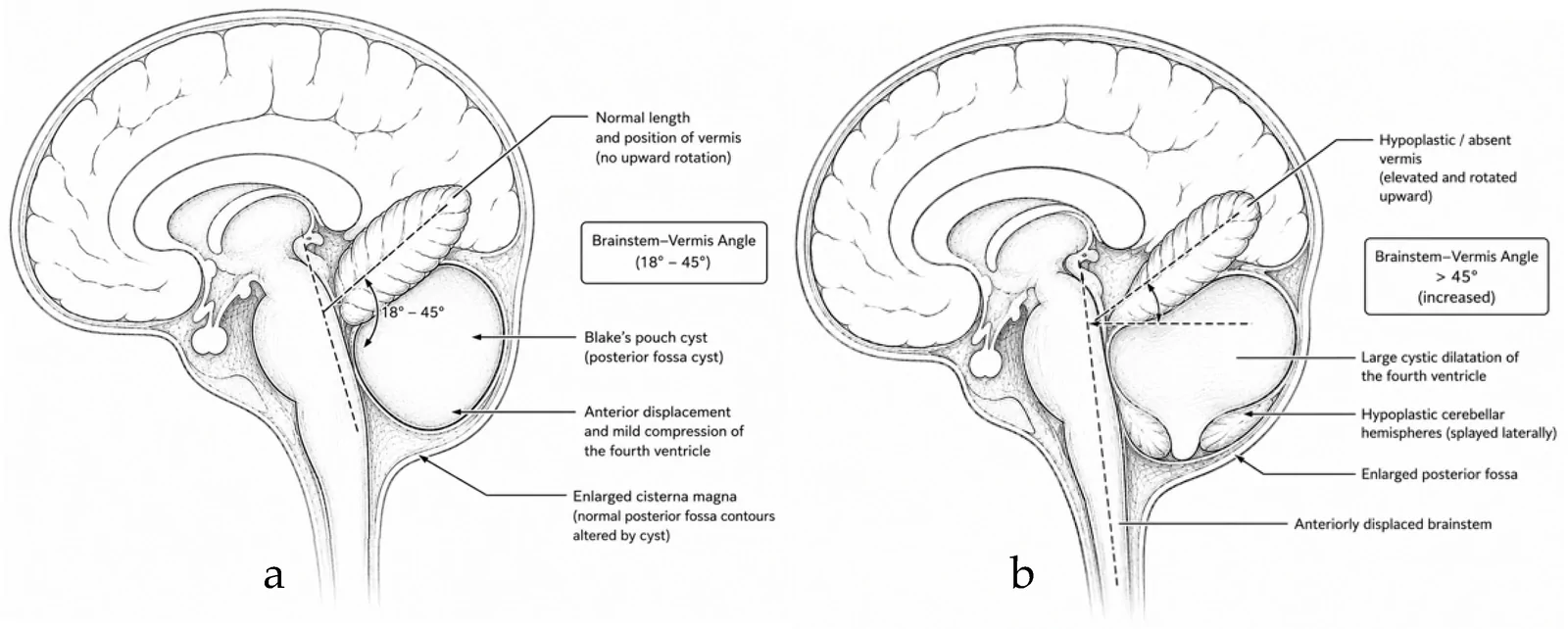
Anatomical sketch of (**a**) Blake’s pouch cyst and (**b**) Dandy–Walker malformation.

**Figure 10:**
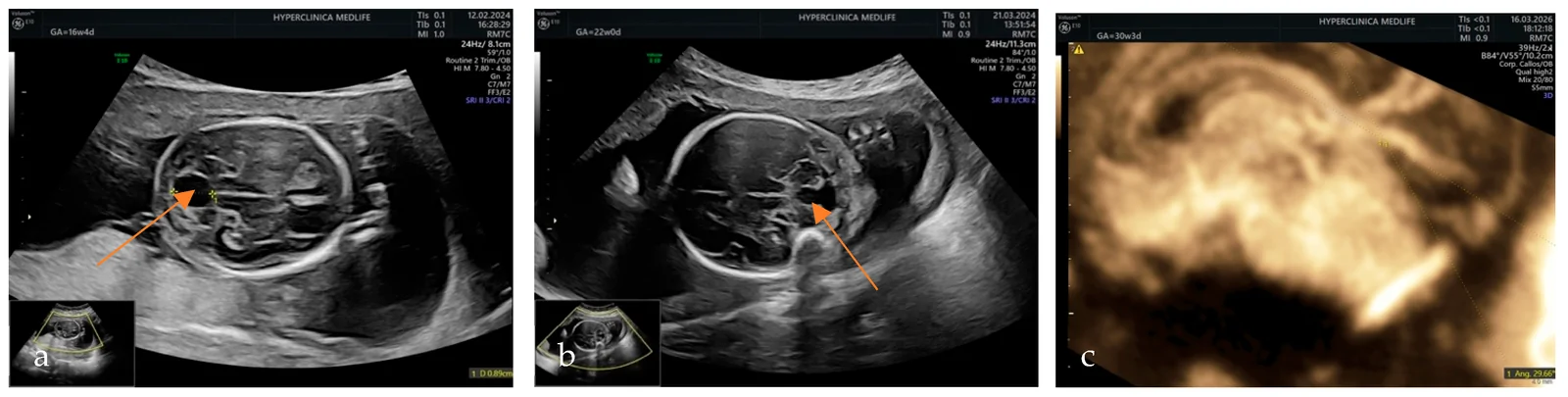
(**a**) BPC on ultrasound at 16 weeks of gestation (orange arrow). (**b**) BPC at 22 weeks of gestation—“keyhole” sign (orange arrow). (**c**) Brainstem–vermis angle measurement.

**Figure 11:**
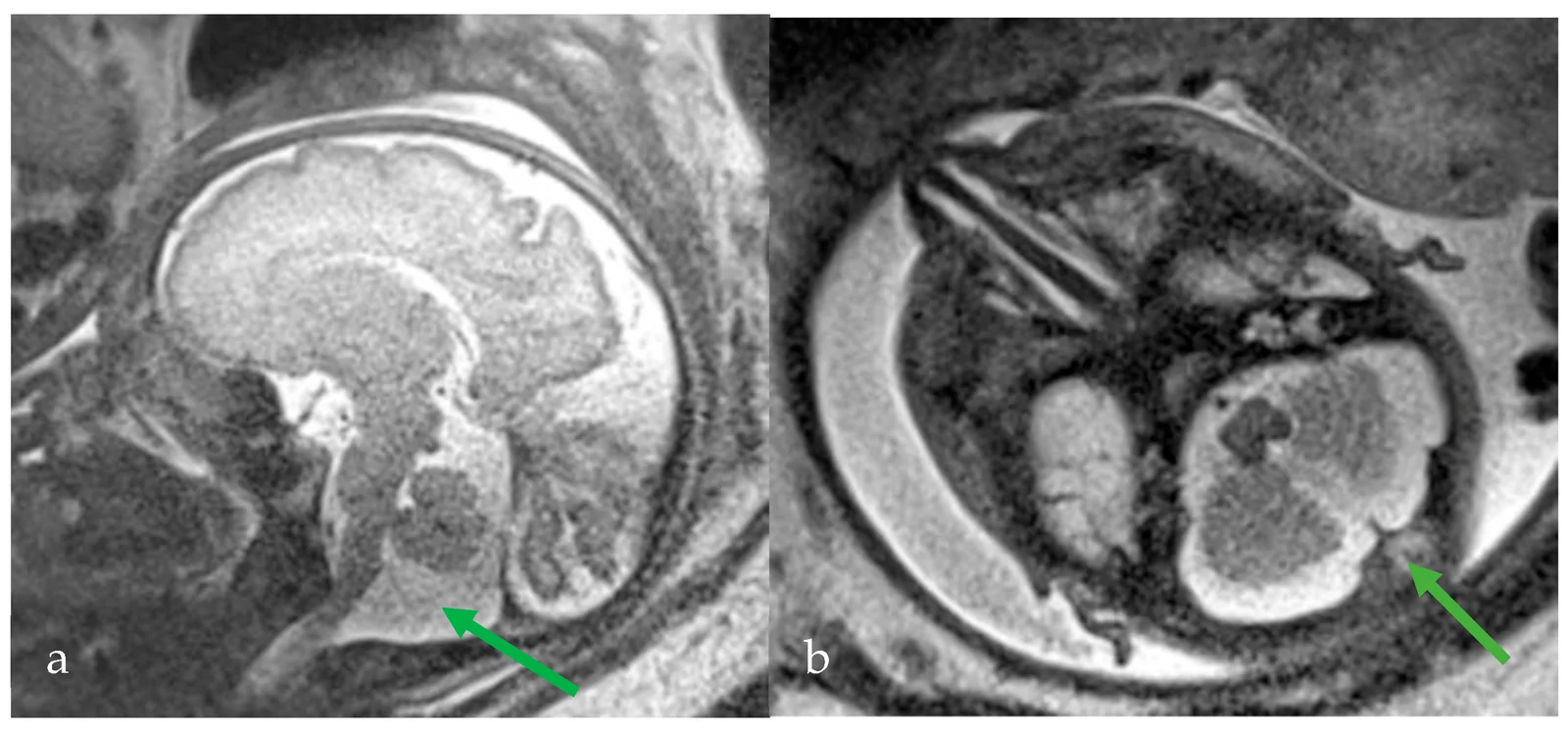
Fetal MRI in a case of BPC at 32 weeks of gestation. (**a**) T2 axial section with posterior cystic enlargement (green arrow). (**b**) Sagittal T2 section at the level of the vermis (green arrow).

**Figure 12:**
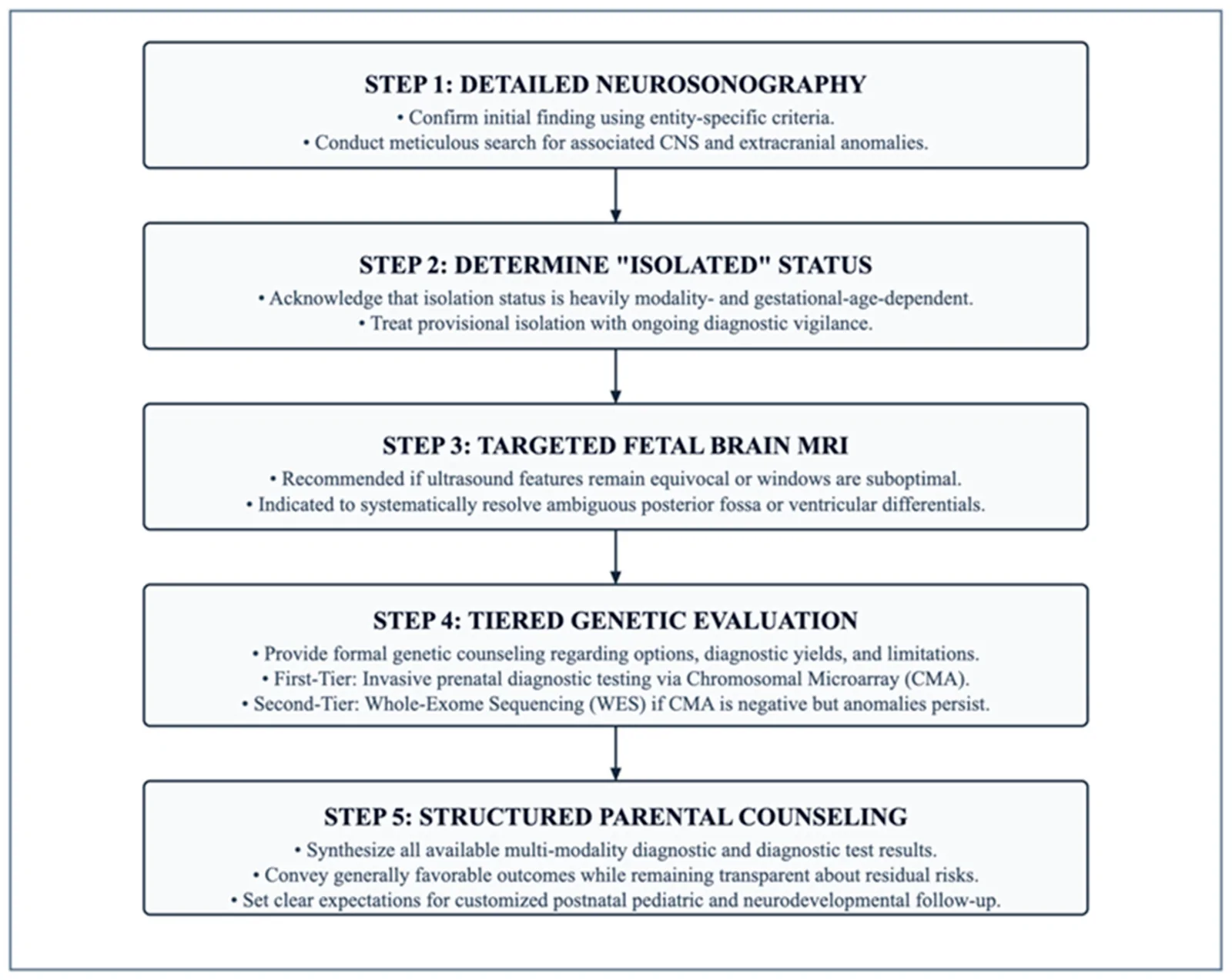
Staged approach to minor abnormalities of the fetal brain on antenatal ultrasound.

## Data Availability

No new data were created or analyzed in this study. Data sharing is not applicable to this article.

## Supplementary Materials

The following supporting information can be downloaded at https://www.mdpi.com/article/10.3390/diagnostics16193144/s1: Table S1: Search strings by database. Last search: 24 August 2026.

## Abbreviations

ACC	Agenesis of the corpus callosum
AD	Atrial diameter
AP	Anteroposterior
BPC	Blake’s pouch cyst
CMA	Chromosomal microarray analysis
CMV	Cytomegalovirus
CNS	Central nervous system
CNV	Copy number variation
cfDNA	Cell-free DNA
CSF	Cerebrospinal fluid
DWC	Dandy–Walker continuum
DWM	Dandy–Walker malformation
ISUOG	International Society of Ultrasound in Obstetrics and Gynecology
MCM	Mega cisterna magna
MRI	Magnetic resonance imaging
MV	Mild ventriculomegaly
NDVA	Non-dilated ventricular asymmetry
NIPT	Non-invasive prenatal testing
PCR	Polymerase chain reaction
SMFM	Society for Maternal-Fetal Medicine
US	Ultrasound
VM	Ventriculomegaly
WAPM	World Association of Perinatal Medicine
WES	Whole-exome sequencing
WGS	Whole-genome sequencing
