# Supplementary material for: Mild Ventriculomegaly, Ventricular Asymmetry, and Posterior Fossa Variants: A Narrative Review of Borderline Fetal Brain Findings
Source: Diagnostics (Basel). 2026 Sep 27;16(19):3144. doi: 10.3390/diagnostics16193144 (PMC13664448; doi:10.3390/diagnostics16193144)
Supplement: Supplementary file 1 [file diagnostics-16-03144-s001.zip › diagnostics-4507573-supplementary.pdf]

Supplementary Table S1. Search strings by database. Last search: 24 August 2026.

Search string	Database	Search date	Results
(fetal[tiab] OR fetus[tiab] OR prenatal[tiab] OR antenatal[tiab]) AND (ventriculomegaly[tiab] OR "ventricular asymmetry"[tiab] OR "mega cisterna magna"[tiab] OR "Blake's pouch"[tiab] OR "posterior fossa"[tiab]) AND ("magnetic resonance imaging"[tiab] OR MRI[tiab] OR "chromosomal microarray"[tiab] OR "exome sequencing"[tiab] OR "genome sequencing"[tiab] OR "congenital infection"[tiab] OR neurodevelopment*[tiab] OR outcome*[tiab] OR prognosis[tiab])	**PubMed/MEDLINE**	25.08.2026	1233
TITLE-ABS-KEY((fetal OR fetus OR prenatal OR antenatal) AND (ventriculomegaly OR "ventricular asymmetry" OR "mega cisterna magna" OR "Blake's pouch" OR "posterior fossa") AND ("magnetic resonance imaging" OR MRI OR "chromosomal microarray" OR "exome sequencing" OR "genome sequencing" OR "congenital infection" OR neurodevelopment* OR outcome* OR prognosis))	**Scopus (Advanced document search)**	25.08.2026	1985
TS=((fetal OR fetus OR prenatal OR antenatal) AND (ventriculomegaly OR "ventricular asymmetry" OR "mega cisterna magna" OR "Blake's pouch" OR "posterior fossa") AND ("magnetic resonance imaging" OR MRI OR "chromosomal microarray" OR "exome sequencing" OR "genome sequencing" OR "congenital infection" OR neurodevelopment* OR outcome* OR prognosis))	**Web of Science Core Collection (Advanced search)**	25.08.2026	1434

Language restriction: English. No date restriction applied. Reference lists of included articles and of ISUOG, SMFM and WAPM guidance documents were hand-searched for additional relevant publications.
